# Advancements in the Regulation of Different-Intensity Exercise Interventions on Arterial Endothelial Function

**DOI:** 10.31083/j.rcm2411306

**Published:** 2023-11-01

**Authors:** Qian-Qian Li, Kai-Rong Qin, Wen Zhang, Xiu-Mei Guan, Min Cheng, Yan-Xia Wang

**Affiliations:** ^1^School of Rehabilitation Medicine, Weifang Medical University, 261053 Weifang, Shandong, China; ^2^School of Health and Life Sciences, University of Health and Rehabilitation Sciences, 266071 Qingdao, Shandong, China; ^3^School of Biomedical Engineering, Faculty of Medicine, Dalian University of Technology, 116024 Dalian, Liaoning, China; ^4^Department of Neurology, Nanjing Drum Tower Hospital Group Suqian Hospital, 223800 Suqian, Jiangsu, China; ^5^School of Basic Medicine Sciences, Weifang Medical University, 261053 Weifang, Shandong, China

**Keywords:** exercise, arterial endothelial function, cardiovascular diseases, vascular diastolic-systolic factors, oxidative stress, inflammatory reaction

## Abstract

Normal-functioning endothelium is crucial to maintaining vascular homeostasis and inhibiting the development and progression of cardiovascular diseases such as atherosclerosis. Exercise training has been proven effective in regulating arterial endothelial function, and the effect of this regulation is closely related to exercise intensity and the status of arterial endothelial function. With this review, we investigated the effects of the exercise of different intensity on the function of arterial endothelium and the underlying molecular biological mechanisms. Existing studies indicate that low-intensity exercise improves arterial endothelial function in individuals who manifest endothelial dysfunction relative to those with normal endothelial function. Most moderate-intensity exercise promotes endothelial function in individuals with both normal and impaired arterial endothelial function. Continuous high-intensity exercise can lead to impaired endothelial function, and high-intensity interval exercise can enhance both normal and impaired endothelial function. In addition, it was demonstrated that the production of vasomotor factors, oxidative stress, and inflammatory response is involved in the regulation of arterial endothelial function under different-intensity exercise interventions. We posit that this synthesis will then provide a theoretical basis for choosing the appropriate exercise intensity and optimize the prescription of clinical exercise for persons with normal and impaired endothelium.

## 1. Introduction

Arterial endothelial cells are located in the innermost layer of the vascular wall and are not only a physical barrier between the blood and the vascular wall but also an important regulator of vascular homeostasis. In addition to its regulation by chemical factors, arterial endothelial cells can respond adaptively to mechanical force signals such as fluid shear stress, circumferential stress, and stretch stress acting on the vessel wall. Then, in response, they can produce a variety of endogenous vasoactive factors such as nitric oxide (NO), prostacyclin, endothelin-1 (ET-1), and vascular cell adhesion molecule-1 (VACM-1), all of which are involved in the regulation of vascular tone, inflammatory response, oxidative stress, and other endothelial functions [1, 2, 3]. Studies have revealed that normal arterial endothelial function is essential for maintaining vascular health, while impaired endothelial function constitutes the initiating factor in the development and progression of cardiovascular diseases [4, 5]. Therefore, delaying or inhibiting cardiovascular diseases by augmenting arterial endothelial function has developed into a key strategy in the prevention and treatment of cardiovascular disease.

Exercise training is an effective method used to prevent and rehabilitate noninvasive cardiovascular diseases [6, 7, 8]. It is generally postulated that moderate-intensity exercise interventions improve arterial endothelial function by inducing an increase in the amplitude and frequency of blood shear stress, promoting endothelial nitric oxide synthase (eNOS) protein expression and NO release, and inhibiting the production of pro-inflammatory factors; whereas high-intensity exercise can lead to oxidative stress and decreased NO bioavailability, resulting in arterial endothelial dysfunction [9, 10, 11, 12, 13]. However, the actions of low-intensity exercise on endothelial function and the effects of different intensity exercise on normal and impaired individuals remain unknown. Therefore, in this paper we review the effects of low-, moderate-, and high-intensity exercise interventions on normal and impaired vascular endothelial functions and their governing molecular biological mechanisms, thus providing a theoretical basis for selecting the most appropriate exercise intensity for disparate populations.

## 2. Evaluation Methods and Indicators of Arterial Endothelial Function

Current indicators of endothelial function in arteries are divided into two main categories: invasive indicator and non-invasive indicator. Endothelial function was measured invasively in earlier studies using coronary angiography; this modality allowed observation of the diastolic response of the vessel after intracoronary injection of the endothelium-dependent vasodilator acetylcholine (ACh). When endothelial function is normal, the addition of ACh induces diastole of the coronary arteries as it stimulates the endothelial cells to secrete nitric oxide. However, when endothelial dysfunction occurs, the coronary arteries are not able to release NO effectively, and local vasoconstriction occurs due to vascular contraction [14]. The principal non-invasive tests currently employed are endothelium-dependent flow-mediated dilatation (FMD) and reactive congestion-peripheral arterial tonometry (PAT). FMD is widely used in cardiovascular clinical trials, and its application is based on high-resolution ultrasonography to assess changes in brachial artery diameter in brachial artery diameter in response to ischemia (typically 5 min). Normal blood vessels, in response to various physiologic and chemical stimuli, usually dilate to modulate blood flow and distribution. After reactive congestion is induced by cuff compression, blood flow to the forearm generates blood shear stress that induces endothelial cells to release NO, and this causes vascular dilatation. When endothelial dysfunction occurs, NO release is reduced, resulting in an abnormal diastolic response of the vascular wall to stimulation and attenuated FMD levels [15, 16]. Maruhashi *et al*. [17] suggested that a value of FMD <7.1% to be the cut-off point for diagnosing endothelial dysfunction. In a study carried out in China, FMD values ranged from 8.29–8.80% in females and 8.34–8.77% in males in healthy chinese children and adolescents [18]. Heiss *et al*. [19] proposed that FMD <6.5% was classified as endothelial dysfunction. At present, there is no specific parameter on the range of reference values for diagnosing endothelial dysfunction. Usually, FMD <6–7% is served as a parameter for diagnosing endothelial dysfunction. PAT is used to calculate the reactive hyperemia index (RHI) by measuring the degree of pulse wave amplitude in the fingertip arteries before and after reactive congestion using a peripheral arterial pressure device. The RHI represents the degree of NO-mediated vasodilatory response and reflects endothelial function, with an RHI <1.67 indicating endothelial dysfunction [20].

In addition to the endothelial function tests described above, arterial endothelial function can also be evaluated by biochemical indicators. These include protein expression levels of the vasodilatory factor NO and vasoconstrictor factor ET-1; eNOS and its phosphorylation level [21]; the oxidative stress products reactive oxygen species (ROS) [12]; the antioxidant enzymes superoxide dismutase (SOD) and glutathione peroxidase (GPX) [22]; and the inflammatory factors C-reactive protein (CRP), monocyte chemoattractant protein-1 (MCP-1), and von Willebrand factor (vWF) [11]. These biochemical indicators are usually used in experimental studies, and rarely served as clinical diagnostic parameters due to their invasive acquisition. At present, FMD is the gold standard for clinical diagnosis of endothelial dysfunction.

## 3. Modulation of Arterial Endothelial Function through Different-Intensity Exercise Interventions

### 3.1 Exercise Intensity and Frequency

Exercise-induced changes in flow shear stress, vascular circumferential stress, and stretch stress can modulate endothelial function, and the effect of this modulation is related to the intensity of exercise. The selection of the appropriate exercise intensity is therefore essential for the maintenance and improvement of endothelial function. Exercise intensity is usually defined in clinical and experimental studies in terms of the percentage of maximal oxygen uptake (${\mathrm{\%VO}}_{\text{2max}}$, maximal oxygen uptake), percentage of maximal heart rate (${\mathrm{\%HR}}_{\text{max}}$), percentage of heart rate in reserve (%HRR, heart rate in reserve), and one-repetition maxmimum (1RM) (for resistance exercise). In resistance exercise, the 1RM that the body can lift in one repetition is used to measure its exercise intensity. In human exercise, low intensity is defined as <40% ${\mathrm{VO}}_{\text{2max}}$ or HRR <64% ${\mathrm{HR}}_{\text{max}}$, and 30–50% 1RM; moderate intensity is defined as 40–60% ${\mathrm{VO}}_{\text{2max}}$ or HRR at 64–76% ${\mathrm{HR}}_{\text{max}}$, and 50–70% 1RM; and high intensity is defined as >60% ${\mathrm{VO}}_{\text{2max}}$ or HRR >76% ${\mathrm{HR}}_{\text{max}}$, and 70–85% 1RM [23]. The grading of exercise intensity often refers to the Bedford exercise protocol for the approximate setting of exercise load in animal experiments, with a running treadmill speed of 8–10 m/min and an incline of 0° for low-intensity exercise; a running treadmill speed of 15–20 m/min and an incline of 5° for moderate-intensity exercise; and a running treadmill speed of >20 m/min and an incline of 10° for high-intensity exercise.

In addition, according to different exercise frequencies, exercise with different intensity is further divided into acute and long-term exercise. Acute exercise refers to a single bout of exercise, and long-term exercise is repetitive bouts of regular exercise. It is worth noting that acute high-intensity exercise includes acute high-intensity continuous exercise and acute high-intensity interval training (HIIT) or high-intensity interval exercise (HIIE). HIIT/HIIE is a form of intermittent exercise that has emerged in recent years, and encompasses the completion of numerous high-intensity exercise in a short period, and is interspersed with low-intensity exercise or rest between two high-intensity exercises [11]. Accordingly, long-term high-intensity exercise contains long-term high-intensity continuous exercise and long-term HIIT/HIIE.

### 3.2 Different Populations with Normal or Impaired Endothelial Function

To analyze the impact of different intensity exercise on persons with normal or impaired endothelial function to provide appropriate exercise intensity for them, the different populations were included in this review. These populations mainly include healthy young people, older adults, postmenopausal women, obese persons and patients with diabetes, hypertension or other cardiovascular diseases. Healthy young people tend to possess normal endothelial function, while impaired endothelial function is commonly associated with the other populations mentioned above. Some healthy elderly men also possess normal endothelial function. Accordingly, animals with normal and impaired endothelial function were included in the review [24, 25, 26]. It should be noted that human and animal experimental data on different intensity exercise regulating endothelial function were searched on PubMed, Web of Science and Google Scholar from 2003 to 2023. 70 pieces of literature were selected to review.

### 3.3 Modulation of Arterial Endothelial Function through a Low-Intensity Exercise Intervention

Extant studies have shown differential results in the modulation of arterial endothelial function through low-intensity exercise (Table 1, Ref. [9, 11, 13, 22, 27, 28]). Goto *et al*. [9] and Birk *et al*. [27] did not uncover any significant changes in FMD levels or forearm blood flow in response to ACh in healthy young adults for either acute exercise session or a long-term low-intensity exercise intervention lasting 12 weeks; this indicated that endothelial function did not undergo a significant improvement. However, Shimizu *et al*. [11] found that RHI levels after 4 weeks of a low-intensity resistance-exercise intervention in healthy elderly people were elevated from 1.8 ± 0.2 vs. 2.1 ± 0.3 and that their vWF levels were reduced, leading to enhanced NO-mediated vasodilation and reduced endothelial cell injury in blood vessels—suggesting that short-term low-intensity exercise improved vascular endothelial function in the elderly. In addition, Merino *et al*. [22] demonstrated that low-intensity walking exercise for 4 consecutive months improved antioxidant capacity and vascular endothelial function in overweight and obese postmenopausal women by augmenting the activity of the antioxidant enzymes SOD (9506 ± 3408 vs. 12,628 ± 2472 μmol/min/grHb) and GPX (1.97 ± 0.51 vs. 2.26 ± 0.77), increasing their small-artery reactive congestion index.

**Table 1.** **Modulation of arterial endothelial function through a low-intensity exercise intervention**.

Research subjects	Exercise program			Changes in endothelial-function test indicators			Literature sources
	Intensity	Duration and frequency of exercise	Forms	Indicators	Values (before vs. after exercsie)	Change	
Healthy young men (n = 10)	25% ${\mathrm{VO}}_{\text{2max}}$	30 min/d, 5–7 times/w, 12 w	Cycling	FBF	5.0 ± 1.4 vs. 4.8 ± 1.0 mL/L	NS	[9]
Healthy young men (n = 10)	50% ${\mathrm{HR}}_{\text{max}}$	One time, 30 min	Cycling	FMD	6.3 ± 2.6 vs. 5.9 ± 2.5%	NS	[27]
Healthy elderly men (n = 20)	20% 1RM	15 min/d, 3 d/w, 4 w	Resistance exercise	RHI	1.8 ± 0.2 vs. 2.1 ± 0.3	(↑) *p <* 0.01	[11]
				vWF	175.7 ± 20.3 vs. 156.3 ± 38.1%	(↓) *p <* 0.05	
Overweight and obese Postmenopausal women (n = 47)		1 h/d, 2 d/w, 4 m	Walking	GPX	9506 ± 3408 vs. 12,628 ± 2472 μmol/min/grHb	(↑) *p <* 0.001	[22]
				saRHI	1.97 ± 0.51 vs. 2.26 ± 0.77	(↑)* p* = 0.043	
Eight-week-old C57BL/6J mice (n = 6)	5 m/min	60 min/d, 6 d/w, 4 w	Treadmill training	The number of EPC	497 ± 10 vs. 534 ± 10 number/mL	(↑) *p * < 0.05	[28]
db/db Sprague-Dawley rats (n = 11)	10 m/min	1 h/d, 6 w	Treadmill training	NO	4.22 ± 1.7 vs. 6.78 ± 2.1 µmol/L	(↑) *p <* 0.05	[13]
				eNOS	9.87 ± 3.5 vs. 14.67 ± 3.8 µmol/L	(↑) *p * < 0.05	
				vWF	vWF decreased by 20.4%	(↓) *p * < 0.05	

NS indicates no significant, ↑ indicates increase, ↓ indicates decrease. ${\mathrm{VO}}_{\text{2max}}$, maximal oxygen uptake; FBF, forearm blood flow; ${\mathrm{HR}}_{\text{max}}$, maximal heart rate; FMD, flow-mediated dilatation; RHI, reactive hyperemia index; vWF, von Willebrand factor; GPX, glutathione peroxidase; EPC, endothelial progenitor cell; NO, nitric oxide; eNOS, endothelial nitric oxide synthase; h/d, hour/day; d/w, day/week; saRHI, small artery reactive hyperemia index; 1RM, one-repetition maxmimum.

The results of experimental animal studies revealed that after 6 weeks of 10 m/min treadmill training in diabetic rats, serum NO levels and eNOS expression levels were elevated and vWF was decreased in the exercise group, proving beneficial to endothelial function; and appeared to prevent and improve diabetic cardiomyopathy [13]. Similarly, blood pressure was significantly reduced in rats suffering from severe hypertension after long-term low-intensity exercise training, and their impaired endothelium-dependent vasodilatory function and insulin sensitivity were also improved [29].

The reasons subserving the production of differential modulatory effects on arterial endothelial function with low-intensity exercise described in the aforementioned studies may have related to whether the human subjects or rats initially possessed healthy arterial endothelial function. As previously mentioned, healthy young people usually possess normal endothelial function, while impaired endothelial function tends to occur in healthy young people, older adults, postmenopausal women, obese persons and patients with cardiovascular diseases. However, whether the initial healthy or impaired endothelial function is a critical factor in different intensity exercise regulating endothelial function requires further study.

### 3.4 Modulation of Arterial Endothelial Function through a Moderate-Intensity Exercise Intervention

A series of studies have shown that acute and long-term moderate-intensity exercise promote arterial endothelial function in healthy individuals as well as in the elderly, hypertensives, diabetics, and patients after myocardial infarction (Table 2, Ref. [9, 21, 28, 30, 31, 32, 33, 34, 35, 36, 37]). Boeno *et al*. [30] ascertained that acute moderate-intensity resistance exercise accelerated vasodilation by increasing nitrites and nitrates (${\mathrm{NO}}_{\text{X}}$) level from 6.8 ± 3.3 to 12.6 ± 4.2 µM and FMD from 12.5 ± 4.10 to 17.2 ± 3.9% in sedentary middle-aged men. In addition, patients undergoing percutaneous coronary intervention (PCI) after acute myocardial infarction showed significant progress in endothelial function after an acute moderate-intensity (50–60% HRR) exercise intervention [31]. Landers-Ramos *et al*. [32] additionally found a significant increase in FMD levels from 10 ± 1.3 to 16 ± 1.4% after moderate-intensity exercise training for 10 days in sedentary older adults following an aerobic exercise intervention at 70% ${\mathrm{VO}}_{\text{2max}}$ for ten consecutive days. Another study comprising hypertensive patients as subjects undergoing long-term exercise at 60–80% HRR exercise intensity for 12 weeks revealed a 1.7 ± 2.8% increase in FMD, and a decrease in ET-1 levels and the related inflammatory factors CRP, MCP-1, and VACM-1 levels; with improvements in blood pressure, inflammation, and endothelial function associated with cardiovascular health [33]. Meta-analysis also showed that long-term exercise training for more than 8 weeks significantly increased overall FMD levels in patients with type II diabetes, and low-to-moderate-intensity exercise augmented FMD levels compared with high-intensity continuous exercise [38, 39]. Most studies [9, 21, 28, 30, 31, 32, 33, 34, 35, 36, 37] have demonstrated that moderate-intensity exercise can ameliorate endothelial dysfunction. However, Shah *et al*. [24] found that prior acute moderate-intensity exercise compared with no exercise did not affect FMD, ET-1 and NO concentration following a high sugar meal in postmenopausal women. A previous study confirmed that impaired endothelial function caused by high sugar intake was restored with acute moderate intensity in young men [40]. Therefore, we speculate the dual disadvantage factors acting on endothelial function, including high sugar and menopause, inhibit the effectiveness of moderate intensity exercise on improving endothelial function in Shah *et al*.’s study [24].

**Table 2.** **Modulation of arterial endothelial function through a moderate-intensity exercise intervention**.

Research subjects	Exercise program			Changes in endothelial-function test indicators			Literature sources
	Intensity	Duration and frequency of exercise	Form	Indicator	Values (before vs. after exercsie)	Change	
Sedentary middle-aged men (n = 11)	50% 1RM	One time, 40 min	Resistance exercise	FMD	12.5 ± 4.10 vs. 17.2 ± 3.9%	(↑) *p* = 0.016	[30]
				${\mathrm{NO}}_{\text{X}}$	6.8 ± 3.3 vs. 12.6 ± 4.2 µM	(↑)* p* = 0.007	
Healthy elderly (n = 11)	70% ${\mathrm{VO}}_{\text{2max}}$	60 min/d, 10 d	Treadmill walking or running	FMD	10 ± 1.3 vs. 16 ± 1.4%	(↑)* p * < 0.05	[32]
Patients who underwent PCI after an acute heart attack (n = 20)	50–60% HRR	One time, 30 min	Aerobic exercise	FMD	increased by 4.9%	(↑)* p *= 0.034	[31]
Patients with acute abdominal aortic aneurysm (n = 22)	40% peak power ouput (PPO)	One time, 27 min	Aerobic exercise	FMD	0.69% vs. 1.73%	(↑) *p <* 0.001	[36]
Patients with type II diabetes mellitus (n = 13)	65–85% ${\mathrm{HR}}_{\text{max}}$	1 h/d, 3 d/w, 12 w	Aerobic and resistance exercise	FMD	7.62 ± 1.2 vs. 9.82 ± 1.0%	(↑) *p * < 0.05	[34]
Healthy young men (n = 8)	50% ${\mathrm{VO}}_{\text{2max}}$	30 min/d, 5–7 times/w, 12 w	Cycling	ACh	13.1 ± 8.8 vs. 19.6 ± 12.7 mL/min per 100 mL tissue	(↑)* p * < 0.05	[9]
Patients with hypertension (n = 42)	60–80% HRR	40–50 min/times, 3 times/w, 12 w	Treadmill running	FMD	7.59 ± 3.36 vs. 9.26 ± 2.93%	(↑) *p* = 0.02	[33]
				${\mathrm{NO}}_{\text{X}}$	9.4 ± 3.7 vs. 13.8 ± 4.6 µmol/L	(↑) *p* = 0.005	
				CRP	2.8 ± 1.7 vs. 1.8 ± 0.8 mg/dL	(↓) *p* = 0.03	
				MCP-1	124.1 ± 60.2 vs. 84.4 ± 41.9 pg/mL	(↓) *p *= 0.009	
				VCAM-1	1387.8 ± 705.2 vs. 1084.8 ± 433.1 pg/mL	(↓) *p* = 0.03	
				ET-1	6.4 ± 1.6 vs. 4.7 ± 0.7 pg/mL	(↓) *p * < 0.001	
Young men with hypertension (n = 18)	40–50% HRR	One time, 40 min	Cycling	NO	65–70.85 µmol/L	(↑) *p * < 0.01	[37]
Five-week-old db/db mouse (n = 8)	5.2 m/min	1 h/d, 5 d/w, 7 w	Wheel training	Mn-SOD		(↑) *p<* 0.05	[21]
				eNOS Ser1117			
Three-month-old SHR (n = 8)	18–20 m/min	60 min/d, 5 d/w, 8 w	Treadmill training	ROS		(↑) *p * < 0.05	[35]
				NO		(↓) *p * < 0.05	
Eight-week-old C57BL/6J mice (n = 6)	10 m/min	60 min/d, 6 d/w, 4 w	Treadmill training	The number of EPC	497 ± 10 vs. 534 ± 10 number/mL	(↑) *p * < 0.05	[28]

↑ indicates increase, ↓ indicates decrease. FMD, flow-mediated dilatation; ET-1, endothelin-1; 8-OHdG, 8-hydroxy-2 deoxyguanosine; NO, Nitric oxide; ROS, reactive oxygen species; ${\mathrm{VO}}_{\text{2max}}$, maximal oxygen uptake; ${\mathrm{HR}}_{\text{max}}$, maximal heart rate; HRR, heart rate in reserve; Ach, acetylcholine; CRP, c-reactive protein; Mn-SOD, mitochondrial manganese superoxide dismutase; eNOS, endothelial nitric oxide synthase; EPC, endothelial progenitor cell; MCP-1, monocyte chemoattractant protein-1; VCAM-1, vascular cell adhesion molecule-1; h/d, hour/day; d/w, day/week; ${\mathrm{NO}}_{\text{X}}$, nitrites and nitrates; 1RM, one-repetition maxmimum.

The effect of an exercise intervention on arterial endothelial function also exerts a significant temporal effect, with the effect on endothelial function diminishing or even disappearing after the end of the exercise. Naylor *et al*. [34] found that 12 weeks of combined aerobic and resistance exercise in adolescent type II diabetic patients produced a significant elevation in brachial artery FMD levels from 7.62 ± 1.2 to 9.82 ± 1.0% while effectively controlling patient blood glucose levels. However, after 12 weeks of discontinuation of training, the data showed that brachial FMD gradually returned to resting levels and those endothelial functional changes gradually disappeared.

The results of experimental animal studies showed that long-term moderate-intensity wheel exercise was observed to normalize diabetes-related endothelial dysfunction and improve insulin sensitivity in a diabetic mouse model, and the results suggested a reversal of type II diabetic endothelial dysfunction by enhancing NO bioavailability through elevated production of mitochondrial manganese superoxide dismutase (Mn-SOD), total eNOS protein, and phospho-eNOS (Ser1177) [21]. Furthermore, Ye *et al*. [35] trained hypertensive rats to run at 18–20 m/min (55–65% ${\mathrm{VO}}_{\text{2max}}$) for 60 min per day, 5 days per week for 8 weeks, and discerned that long-term moderate-intensity exercise prevented hypertension-related endothelial ultrastructural remodeling and endothelial dysfunction by alleviating oxidative stress and enhancing NO-mediated diastolic response in the mesenteric arteries of hypertensive rats. In another study, the authors established a mouse model of hypoxia-induced endothelial cell injury and found that moderate-intensity exercise for 4 weeks augmented the release of circulating exosomes derived from endothelial progenitor cells (EPCs) and elevated the expression of exosome miR-126. This exercise reduced the apoptotic rate of endothelial cells, thereby protecting and enhancing endothelial cell function [28].

In conclusion, most acute and long-term moderate-intensity exercise training effectively improve endothelial function in different populations, but their effects on endothelial function have certain time limitations. Therefore, both healthy individuals with normal endothelial function and those with endothelial dysfunction need to maintain an effective exercise regimen to improve endothelial function through long-term exercise. In addition, double or multiple unfavorable factors acting on endothelial function may be able to weaken or inhibit the improvement of moderate intensity exercise on endothelial function. Thus, people with endothelial dysfunction who expect to obtain a beneficial effect of exercise need to minimize the influence of adverse factors, such as reducing high sugar intake.

### 3.5 Modulation of Arterial Endothelial Function through a High-Intensity Exercise Intervention

Some studies suggest that one bout or repetitive bouts of sustained high-intensity exercises cause oxidative stress and the development of cellular inflammatory responses, leading to impaired endothelial function, while others suggest that repetitive bouts of HIIT/HIIE exerts a positive effect on the regulation of arterial endothelial function (Table 3, Ref. [9, 12, 27, 30, 35, 41, 42, 43, 44, 45, 46]). Birk *et al*. [27] described a significant diminution in FMD levels from 6.6 ± 1.6 to 3.6 ± 2.2% after acute high-intensity continuous exercise, and Nyborg *et al*. [41] discerned that athletes participating in the high-intensity continuous Norwegian triathlon (an all-around sport) exhibited a transient 5.6% decrease in FMD response immediately after the race, with a corresponding drop in L-arginine (NO precursor) levels and a rise in the levels of the endothelial inflammatory markers E-selectin, vascular cell adhesion molecule-1 (VCAM-1), and intercellular cell adhesion molecule-1 (ICAM-1). This implied that the inflammatory response induced by overly intense exercise led to endothelial dysfunction. Other studies have shown that long-term sustained high-intensity exercise also impaired endothelium-dependent vasodilation and reduced NO synthesis and secretion by reducing antioxidant levels and increasing oxidative stress. For example, Goto *et al*. [9] subjected healthy young men to intense cycling training at 75% ${\mathrm{VO}}_{\text{2max}}$ for 12 weeks, and found that the subjects’ blood reflected an increase in ${\mathrm{8-hydroxy-2}}^{\prime }$-deoxyguanosine (8-OHdG) from 6.7 ± 1.1 to 9.2 ± 2.3 ng/mL and malondialdehyde-low density lipoprotein (MDA-LDL) from 69.0 ± 19.5 vs. 82.4 ± 21.5 U/L, neither of which was conducive to endothelium-dependent vasodilation. However, HIIT/HIIE with alternating high- and low-exercise intensities was shown to generate a significantly beneficial effect on endothelial function. HIIT was found to improve vascular endothelial function in healthy older adults in the short term, and to reduce the risk of cardiovascular disease to a greater extent than with moderate-intensity continuous exercise, positively affecting the vascular system [42]. Jo *et al*. [43] conducted a comparative exercise intervention between long-term HIIT and moderate-intensity continuous exercise for 8 weeks in 34 patients with hypertension syndrome, and their results suggested that FMD was significantly improved by 6.1% after exercise in both groups, while NO and EPC expression in patients in the HIIT group rose after the intervention. However, there was no change after moderate-intensity continuous exercise.

**Table 3.** **Modulation of arterial endothelial function through a high-intensity exercise intervention**.

Research subjects	Exercise program			Changes in endothelial-function test indicators			Literature sources
	Intensity	Duration and frequency of exercise	Forms	Indicators	Values (before vs. after exercsie)	Change	
Healthy young men (n = 10)	85% ${\mathrm{HR}}_{\text{max}}$	One time, 30 min	Cycling	FMD	6.6 ± 1.6 vs. 3.6 ± 2.2%	(↓) *p * < 0.05	[27]
Young men (n = 9)	extreme sports	One time	Ironman triathlon	FMD	8.7 vs. 3.2%	(↓) *p * < 0.05	[41]
Sedentary middle-aged men (n = 11)	80% 1RM	One time, 40 min	Resistance exercise	ET-1	20.02 ± 2.2 vs. 25.4 ± 2.1 pg/mL	(↓) *p *= 0.004	[30]
Healthy young men (n = 8)	75% ${\mathrm{VO}}_{\text{2max}}$	30 min/d, 5–7 times/w, 12 w	Cycling	8-OHdG	6.7 ± 1.1 vs. 9.2 ± 2.3 ng/mL	(↑) *p * < 0.05	[9]
				MDA-LDL	69.0 ± 19.5 vs. 82.4 ± 21.5 U/L	(↑) *p * < 0.05	
Patients with hypertensive metabolic syndrome (n = 17)	40% HRR 5 min, 60% HRR 5 min, 80% HRR5 min, recovery 40% HRR 5 min (HIIT)	3 d/w, 8 w	Treadmill running	FMD	6.5 ± 4.2 vs. 12.6 ± 6.0%	(↑) *p * < 0.05	[43]
				${\mathrm{NO}}_{\text{X}}$	38.5 ± 21.41 vs. 50.89 ± 20.92 mmol/L	(↑) *p * < 0.05	
Healthy elderly (n = 12)	100% PPO, recovery 15 s, 2 × 20 min/time (HIIT)	3 d/w, 6 w	Cycling	FMD	4.8 ± 1.8 vs. 6.7 ± 1.3%	(↑) *p * < 0.001	[42]
Patients with type 1 diabetes (n = 12)	60% ${\mathrm{HR}}_{\text{max}}$ 5 min, 85% ${\mathrm{HR}}_{\text{max}}$ 1 min × 6 times, 50% ${\mathrm{HR}}_{\text{max}}$ 4 min (HIIT)	40 min/d, 3 d/w, 8 w	Cycling	FMD	5.7 ± 5.0 vs. 11.2 ± 5.4%	(↑) *p * < 0.05	[46]
C57BL/6 mice (n = 6)	Started at 5 m/min and increased by 4 m/min every 10 min until exhaution	One time	Treadmill training	NO		(↓) *p * < 0.01	[44]
Three-month-old SHR (n = 8)	26–28 m/min	60 min/d, 5 d/w, 8 w	Treadmill training	ROS		(↑) *p * < 0.05	[35]
				NO		(↓) *p <* 0.05	
Ten-week-old SHR rats (n = 24)	80% ${\mathrm{VO}}_{\text{2max}}$	1 h/d, 5 d/w, 6 w	Treadmill training	ROS		(↑)* p <* 0.05	[12]
				NO		(↓) *p <* 0.05	
Eight-week-old Zucker rats (n = 12)	10 m/min (3 min) and 18 m/min (4 min) alternating 6 sets	5 d/w, 10 w	Treadmill training	SOD	5.82 ± 0.74 vs. 7.13 ± 0.51 µmol·$\min^{-\,1}$$g^{-\,1}$	(↑) *p * < 0.05	[45]
				GPx	2.09 ± 0.90 vs. 3.29 ± 1.38 µmol·$\min^{-\,1}$$g^{-\,1}$	(↑) *p <* 0.05	

↑ indicates increase, ↓ indicates decrease. ${\mathrm{HR}}_{\text{max}}$, maximal heart rate; FMD, flow-mediated dilatation; ET-1, endothelin-1; 8-OHdG, 8-hydroxy-2 deoxyguanosine; HRR, heart rate in reserve; HIIT, high-intensity interval training; MDA-LDL, malondialdehyde-low density lipoprotein; NO, nitric oxide; ROS, reactive oxygen species; ${\mathrm{VO}}_{\text{2max}}$, maximal oxygen uptake; SOD, superoxide dismutase; GPx, glutathione peroxidase; 1RM, one-repetition maximum; PPO, peak power ouput; h/d, hour/day; d/w, day/week; ${\mathrm{NO}}_{\text{X}}$, nitrites and nitrates.

Relevant experimental studies with animal models have shown that acute and long-term sustained high-intensity exercise leads to impaired endothelial function. Przyborowski *et al*. [44] ascertained that reduced NO production and elevated superoxide anion levels in mice after acute progressive-to-exhaustive exercise approached basal levels after 4 hours of recovery. Other investigators reported that long-term high-intensity continuous exercise increased oxidative stress levels in spontaneously hypertensive rats, leading to eNOS uncoupling, excess ROS production, and attenuated NO bioavailability - which, in turn, adversely affected endothelial function [12, 35]. In the study by Groussard *et al*. [45], SOD and GPx activities in Zucker obese rats rose after 10 weeks of HIIT exercise intervention, improving endothelial function by enhancing antioxidant-defense capabilities.

In summary, high-intensity exercise provokes specific changes to the regulation of arterial endothelial function. Acute or long-term high-intensity sustained exercises can cause inflammatory responses and oxidative stress, reduce NO bioavailability, and adversely affect endothelial function. Acute or long-term HIIT/HIIE, however, enhances arterial endothelial function; and, therefore, those individuals with insufficient exercise time can select HIIT/HIIE. However, because HIIT/HIIE requires several high-intensity exercises in a short period, strict control is needed in exercise intensity, exercise interval time, and exercise frequency to prevent the occurrence of adverse cardiovascular events.

## 4. Potential Mechanisms by which Exercise of Different Intensity Modulates Endothelial Function in Arteries

### 4.1 Exercise Regulates Arterial Endothelial Function by Regulating Vasomotor Factors

As a vasodilator derived from endothelial cells, NO is catalyzed by activated eNOS to metabolize L-arginine. NO regulates vascular tone and also inhibits platelet aggregation and leukocyte adhesion, and is an important indicator of arterial endothelial function [47]. ET-1 is an active peptide secreted by endothelial cells and exerts a robust vasoconstrictive effect. Vascular endothelial dysfunction, then, is associated with a decrease in NO secretion as well as an increase in ET-1 secretion. Studies have shown that mechanical forces (including blood shear stress, circumferential stress, and stretch stress) are important physiologic regulators of the production of both NO and ET-1 in endothelial cells, with blood shear stress being the most important mechanical force stimulus regulating vascular endothelial function [3]. Acute and long-term moderate-intensity exercise and acute HIIE intervention augment perfusion, alter hemodynamic signaling, and induce the upregulation of the phosphatidylinositol 3-kinase/protein kinase B (PI3K/AKT) pathway by increasing the frequency and amplitude of shear stress acting on the eNOS Ser1177 phosphorylation at the vascular wall. Furthermore, this activity activates and increases endogenous NO bioavailability and reduces ET-1 production, thereby improving vascular tone and arterial endothelial function [48, 49, 50, 51]. In contrast, acute high-intensity sustained exercise can uncouple eNOS, leading to a further drop in NO production as well as an elevation in ET-1 concentrations [30, 33]. In addition, acute HIIT exercise mediates increased levels of adipocytokine C1q/tumor necrosis factor-related protein 9 (CTRP9), which may benefit endothelial function in obese individuals by promoting eNOS phosphorylation [52].

### 4.2 Exercise Regulates Arterial Endothelial Function by Modulating Oxidative Stress

Oxidative stress is a pathophysiologic state that results from an imbalance between the oxidative and antioxidant systems and arises when redox homeostasis within an organism is damaged. Dysregulation of redox homeostasis occurs when the production of oxidants such as ROS (including superoxide anion, hydrogen peroxide, and hydroxyl radical) exceeds the production of antioxidants such as superoxide dismutase and glutathione, leading to impaired vascular endothelial function [53]. Studies have shown that low levels of ROS are conducive to maintaining normal cellular homeostasis and blood vessel function, while overproduction of ROS leads to oxidative stress reactions that then lead to the development and progression of cardiovascular diseases. Long-term low-to-moderate-intensity exercise increases the expression of the body’s antioxidant enzymes SOD and GPX, improves antioxidant enzyme defense systems, maintains vascular homeostasis, and reduces the risk of cardiovascular disease by augmenting antioxidant capacity as well as by reducing the overexpression of oxidative enzymes (e.g., reduced nicotinamide adenine dinucleotide phosphate-reduced oxidase and xanthine oxidase [35, 54]). In addition, researchers have identified a mitochondrial inner membrane uncoupling protein 2 (UCP2) that is an important negative regulator of ROS production. Furthermore, they have found that the long-term moderate-intensity exercise intervention upregulated UCP2 expression via the peroxisome proliferator-activated receptor gamma coactivator-1 alpha (PGC1α)/peroxisome proliferator-activated receptor-delta (PPAR-δ) pathway and thereby down-regulated ROS and increased eNOS expression; this, in turn, mitigated metabolic disorders concerning NO bioavailability to alleviate endothelial dysfunction [35, 55, 56]. However, excessive and sustained acute and long-term high-intensity sustained exercise can cause substantial ROS production, induce vascular remodeling, and alter normal physiologic processes, thereby reducing intracellular NO bioavailability and further exacerbating endothelial dysfunction and injury [48, 57, 58].

### 4.3 Exercise Regulates Arterial Endothelial Function by Modulating the Inflammatory Response

Inflammatory responses can induce arterial endothelial dysfunction that constitutes an important trigger for atherosclerosis and structural changes in arteries. The exercise intervention effectively suppresses the inflammatory response, and long-term low-to-moderate-intensity exercise reduces the expression of pro-inflammatory proteins, such as CRP, MCP-1, tumor necrosis factor-alpha (TNF-α) , ICAM-1, and VCAM-1, while increasing the expression levels of anti-inflammatory factors such as IL-4 and IL-10, thereby rectifying arterial endothelial function [33, 59]. Hong *et al*. [56] found that the long-term moderate-intensity exercise mitigated coronary endoplasmic reticulum stress-related endothelial dysfunction by reducing endoplasmic reticulum stress and thioredoxin-interacting protein (TXNIP)/nucleotide-binding and oligomerization (NACHT), leucine-rich repeat (LRR), and N-terminal pyrin domain (PYD) domains-containing protein 3 (NLRP3) inflammatory vesicle expression. In contrast, long-term high-intensity sustained exercise increased the levels of inflammatory factors such as IL-6, creating an inflammatory response [60].

### 4.4 Exercise Regulates Vascular Endothelial Repair and Regeneration

EPCs can differentiate into endothelial cells that are involved in mediating the repair of endogenous vascular endothelial injury and that play a key role in maintaining the structural and functional integrity of the vascular endothelium [61]. Long-term Exercise can increase the number of EPCs, increase the activity of eNOS, enhance the bioavailability of NO, regulate endothelial repair in angiogenesis, and prevent endothelial dysfunction by targeting EPCs [62]. Acute and long-term moderate-intensity aerobic or resistance exercise affects the mobilization of EPCs by augmenting related pro-angiogenic factors such as vascular endothelial growth factor (VEGF),stromal cell-derived factor-1, hypoxia-inducible factor-1, and matrix metalloproteinase-9 to enhance endogenous endothelial repair, which, in turn, repairs and maintains the vascular cytoarchitecture [63, 64, 65].

### 4.5 Exercise Regulates Arterial Endothelial Function by Regulating Exosomes

The release of a range of bioactive molecules in exercise via extracellular vesicles has been identified as a novel phenomenon in mediating intercellular communication that promotes beneficial effects in many systems *in vivo*, and the exercise intervention can induce the release of exosomes from a variety of tissues and thereby improve endothelial function. Exosomes are nano-sized tiny extracellular vesicles that contain proteins, lipids, nucleotides, and other biologically active substances; and these target endothelial cells through direct lipid membrane fusion, receptor-ligand interactions, macropinocytosis, endocytosis, and other pathways to regulate cellular behavior and mediate biological effects. These actions then promote vascular neogenesis, the regulation of vasoconstriction and diastole, and the inhibition of apoptosis and other regulatory endothelial functions [66]. Exercise-induced exosome secretion provides a novel and direct endogenous cardioprotective effect that is closely related to the advancement of intercellular information exchange [67] by exercise, the activation of the sprouty related EVH1 domain containing 1 (SPRED1)/VEGF signaling pathway [28], and increased ${\mathrm{Ca}}^{2+}$ release [68]. Exercise-induced circulating exosomes are thought to manifest a positive regulatory role in mediating signaling processes associated with adaptive responses to exercise, and both acute exercise and long-term chronic exercise exert beneficial actions on endothelial function and protect cardiovascular health by promoting the expression of exosomes and their contents (miRNAs and proteins)-including miRNA-126 and miRNA-342-5P, which enhance intercellular communication [28, 66, 69]. Ma *et al*. [28] demonstrated that long-term low- and moderate-intensity exercise reduced endothelial cell apoptosis and improved endothelial function by increasing miR-126 expression in circulating exosomes, which in turn activated the downstream sprouty related EVH1 domain containing 1 (SPRED1)/VEGF-signal-transduction pathway. Another study revealed that plasma exosomes secreted by mice after 2 weeks of moderate-intensity wheel exercise significantly enhanced endothelial cell migration and angiogenesis compared with sedentary mice and that exosomal SOD3 was important in angiogenesis [70].

## 5. Conclusions

**Fig. 1.**
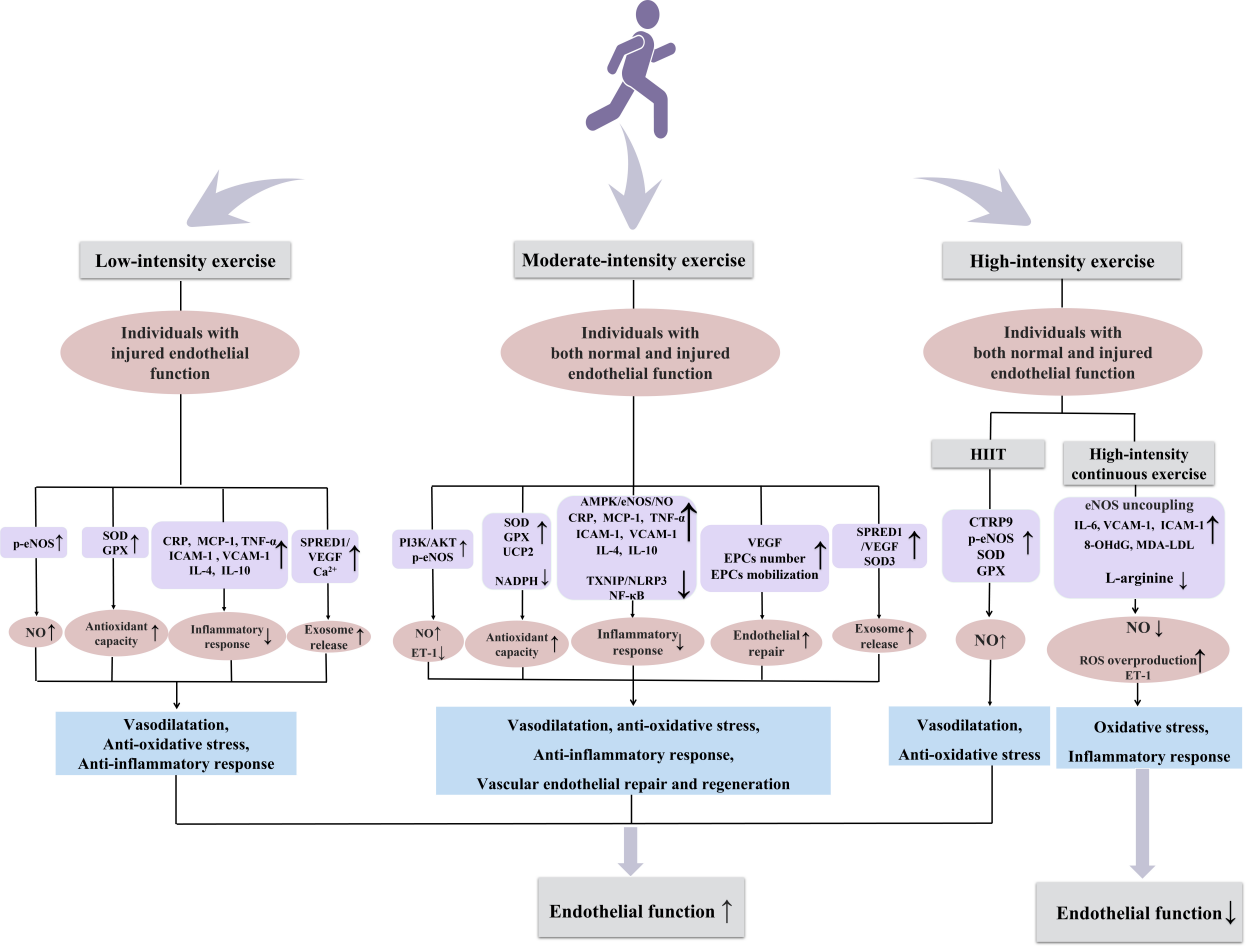
**Potential mechanisms by which exercise of different intensity modulates endothelial function in arteries**. eNOS, endothelial nitric oxide synthase; NO, nitric oxide; GPX, glutathione peroxidase; SOD, superoxide dismutase; CRP, c-reactive protein; MCP-1, monocyte chemoattractant protein-1; TNF-α, tumor necrosis factor-alpha; ICAM-1, intercellular adhesion molecule 1; VCAM-1, vascular cell adhesion molecule-1;IL-4, interleukin-4; IL-10, interleukin-10; SPRED1, sprouty related EVH1 domain containing 1; VEGF, vascular endothelial-derived growth factor ; ET-1, endothelin-1; UCP2, uncoupling protein 2; AMPK, adenosine monophosphate-activated protein kinase; HIIT, high-intensity interval training; EPCs, endothelial progenitor cells; CTRP9, c1q/tumor necrosis factor-related protein 9; IL-6, interleukin-6; 8-OHdG, 8-hydroxy-2 deoxyguanosine; MDA-LDL, malondialdehyde-low density lipoprotein; PI3K/AKT, phosphatidylinositol 3-kinase/protein kinase B.

Exercise applied as a tool in noninvasive active health and cardiovascular disease rehabilitation can effectively modulate arterial endothelial function and thus prevent the onset and development of cardiovascular disease [71]. Through a systematic review of the regulation of different intensities of exercise on endothelial function in disparate populations, we ascertained that low-intensity exercise improved arterial endothelial function in individuals with impaired but not normal endothelial function, while most moderate-intensity exercise and HIIT enhanced endothelial function in both normal and impaired individuals. However, it is unclear as to which is better for those with impaired endothelial function, and systematic and comprehensive studies are, therefore, sorely needed. It is now generally accepted that high-intensity sustained exercise leads to oxidative stress and thus impaired endothelial function and that HIIT improves endothelial function. However, the safety of HIIT in individuals with cardiovascular disease requires further elucidation. Vasodilator production, oxidative stress, inflammatory response, angiogenesis, and exosome secretion have also been shown to be involved in the regulation of endothelial function at different exercise intensities (Fig. 1). Whether there are other potential mechanisms involving in the aforementioned responses necessitates further examination. In addition, we expect that in the near future, the beneficial effects of exercise can be replaced or partly replaced by modulating the abovementioned mechanisms or corresponding signal pathways, which is certainly heartening.

## Abbreviations

Ach, acetylcholine; CTRP9, c1q/tumor necrosis factor-related protein 9; CRP, c-reactive protein; ET-1, endothelin-1; eNOS, endothelial nitric oxide synthase; EPCs, endothelial progenitor cells; FMD, flow-mediated dilatation; GPX, glutathione peroxidase; HIIT, high-intensity interval training; ${\mathrm{HR}}_{\text{max}}$, maximal heart rate; HRR, heart rate in reserve; Mn-SOD, mitochondrial manganese superoxide dismutase; MDA-LDL, malondialdehyde-low density lipoprotein; NO, nitric oxide; PPO, peak power ouput; PAT, peripheral arterial tonometry; PI3K/A, phosphatidylinositol 3-kinase/protein kinase B; PCI, percutaneous coronary intervention; RHI, reactive hyperemia index; ROS, reactive oxygen species; SOD, superoxide dismutase; UCP2, uncoupling protein 2; VCAM-1, vascular cell adhesion molecule-1; vWF, von Willebrand factor; ${\mathrm{VO}}_{\text{2max}}$, Maximal oxygen uptake; 8-OHdG, ${\mathrm{8-hydroxy-2}}^{\prime }$-deoxyguanosine.
